# Prevalence of severe and moderate anthropometric failure among children in India, 1993–2021

**DOI:** 10.1111/mcn.13751

**Published:** 2024-12-04

**Authors:** Menaka Narayanan, Omar Karlsson, Akhil Kumar, Thomas W. Pullum, Rockli Kim, S. V. Subramanian

**Affiliations:** ^1^ Columbia University New York City New York USA; ^2^ Duke Population Research Institute Durham North Carolina USA; ^3^ Faculty of Arts and Sciences University of Toronto Toronto Ontario Canada; ^4^ The Demographic and Health Surveys Program, ICF Rockville Maryland USA; ^5^ University of Texas at Austin Austin Texas USA; ^6^ Division of Health Policy and Management, College of Health Science Korea University Seoul South Korea; ^7^ Interdisciplinary Program in Precision Public Health, Department of Public Health Sciences Graduate School of Korea University Seoul South Korea; ^8^ Harvard Center for Population and Development Studies Cambridge Massachusetts USA; ^9^ Department of Social and Behavioral Sciences Harvard T. H. Chan School of Public Health Boston Massachusetts USA

**Keywords:** 0–2 years, child anthropometry, Social Determinants of Health, states of India, stunting, underweight, wasting

## Abstract

Though child anthropometric failure (CAF) is a persistent problem in India, previous studies have often neglected state‐level variance and aggregated moderate and severe CAF categories. This study addresses this gap by examining moderate and severe malnutrition across India's states and union territories (UTs) from 1993 to 2021. Data of children under 2 years old from five waves of National Family Health Surveys, a representative cross‐sectional survey of Indian households, were analysed. Outcomes included prevalence of moderate and severe stunting, underweight and wasting, as per the 2006 World Health Organization growth standards. Percentage prevalence and standardized absolute change (SAC) were calculated nationally and by region for each wave. From 1993 to 2021, there was a notable reduction in the nationwide prevalence of moderate stunting, underweight and wasting, with rates dropping from 20% to 16%, 23% to 18%, and 15% to 12%, respectively. Severe stunting and underweight declined considerably from 23% to 16% and 18% to 11%, respectively; severe wasting marginally increased from 8% to 9%. From 2016 to 2021 moderate underweight was noted to have the highest SAC across all regions, although 15 regions saw an increase in the prevalence of moderate underweight. In the 2016–2021 period, severe wasting has increased in 13 of the 36 regions. While there has been a nationwide reduction in most indicators of CAF since 1993, the rate and direction of change vary widely among states and UTs and between moderate and severe categories within each of the states and UTs. Understanding these patterns of change can direct context‐specific interventions for improving child nutrition and health. A greater focus on reducing severe wasting, which has increased since 1993, is also crucial.

## Key messages

- Since 1993, moderate anthropometric failure has broadly seen a reduction in 0–2‐year children, with the prevalence of moderate stunting decreasing by 4.5%, the prevalence of moderate underweight decreasing by 5.9% and the prevalence of moderate wasting decreasing by 2.7%.
- While severe stunting and severe underweight have declined considerably (6.3% and 7.3%, respectively) since 1993, severe wasting has slightly increased from by 1%.
- There is substantial variation in the regional trends of anthropometric failure. For example, twelve regions saw an annual average increase of moderate underweight in the 2016–2021 period even though there was an annual average reduction when considering all of India.
- Severe wasting has increased between 2016 and 2021 in 13 regions.

## Graphical abstract

There has been a notable reduction in moderate and severe forms of child anthropometric failure in India since 1993. However, the rate and direction of change vary widely among states and union territories (UTs) and between moderate and severe categories within each of the states and UTs.

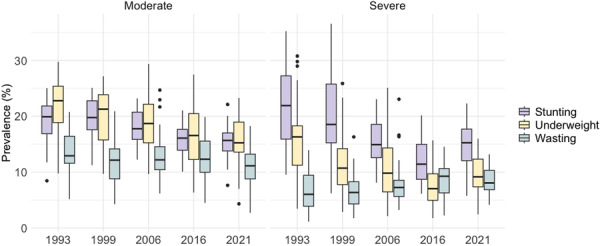

## 1 INTRODUCTION

Proper nutrition is essential for the growth and development of children. Poor nutrition during early years increases the risk of early death and negatively impacts motor, cognitive and behavioural development, leading to detrimental long‐term effects like lower educational achievement, health and productivity (Martorell, 1999). Reducing child undernutrition is a global priority and the United Nations' Sustainable Development Goal 2 (SDG2) focuses on Zero Hunger by 2030 (United Nations Development Program, 2022).

Anthropometric measures are commonly used proxies for describing child undernutrition as well as overweight and obesity (Heemann et al., 2021). Moderate stunting (low height growth), underweight (low weight gain) and wasting (thinness) are defined as height‐for‐age, weight‐for‐age and weight‐for‐height, respectively, between 2 and 3 standard deviations (or z‐scores) below the median of 2006 WHO growth standard (World Health Organization, 2006; World Health Organization, 2019). Children falling more than three standard deviations below the median are considered to suffer from severe stunting, underweight and wasting, respectively.

India, which is home to 19% of the world's children, has much to tackle in the realm of child health (Chandrakant, 2008). India comprises 36 geographic regions (including states, administrative regions and union territories) that are governed by the central government with or without their own legislatures. Much of the health care system and programming are administered at a subnational level, with the central government responsible for providing policy direction and overseeing national initiatives (Baru et al., 2010). There have been deliberate efforts to improve child nutrition as measured by anthropometric outcomes, through national‐ and state‐led efforts (Balarajan & Reich, 2016; Jose & Hari, 2015).

Previous research has reported persistent poor anthropometric outcomes in India, as well as wide disparities across states (Karlsson et al., 2021). However, these studies generally used only the moderate threshold when studying stunting, underweight and wasting, without considering severe anthropometric failure separately (Hemalatha et al., 2020). Presenting data on both moderate and severe anthropometric failure is important to build interventions targeting high‐risk groups, as well as to assess whether such existing interventions have been effective. In this study, we examined trends in both moderate and severe stunting, underweight and wasting between 1993 and 2021 using the most recently available data and harmonization techniques and compared progress within and across India's 28 states and 8 Union Territories.

## 2 METHODS

### 2.1 Data

This study pulls from the National Family Health Survey (NFHS), a nationally representative survey of households in India, wherein families are surveyed on various areas of their lives including the nutrition of their children (Bhat & Zavier, 1999). Given its granularity of household and individual‐level data, the NFHS is often used to convey trends at the state and district level (Ganguly & Unisa, 2010). We specifically used the data fields for state/UT of residence, child age, child height‐for‐age, weight‐for‐age and weight‐for‐height z‐scores—which measure the standard deviation from the growth trajectory of healthy children according to the 2006 WHO growth standard—to estimate the prevalence of stunting, underweight and wasting, respectively. When collecting the height and weight data, measurements of children were taken using standardized procedures consistent across surveys—a digital scale was used to measure weight and a portable measuring board was used to measure height (ICF International, 2012). The five waves of surveys used were: 1992–93, 1998–99, 2005–06, 2015–16 and 2019–21. Each survey is identified in these findings by its end year. This data is available for download at https://dhsprogram.com/.

### 2.2 Study population

This study limited analysis to children between the ages of 0 and 23 months (inclusive). Raw data was pooled from all five survey years, excluded children outside the age range of 0 to 23 months, and then filtered out children with z‐scores that were null due to missing height or weight data. Notably, height data was missing in the 1993 survey for Andhra Pradesh, Chhattisgarh, Himachal Pradesh, Madhya Pradesh, Sikkim, Tamil Nadu, Telangana and West Bengal. Implausible values for z‐scores were filtered out, defined as below ‐6 or above 5 for underweight, below ‐6 or above 6 for stunting and below ‐5 or above 5 for wasting (Assaf et al., 2015). Final analytic samples are listed in Supporting Information: Table S1.

### 2.3 Outcome

For each metric, denominators were defined by taking the full sample of children aged 0–23 months with a valid z‐score for that metric. For each metric, the numerators were defined as the children from the pool of denominators who met the anthropometric failure criteria (i.e., with z‐scores between 2 and 3 standard deviations below the 2006 WHO standard for moderate or more than three standard deviations below the 2006 WHO standard for severe anthropometric failure).

### 2.4 Constructing comparable state estimates

Due to the way state and union territory (UT) delineations have changed since 1993, we chose a geographic harmonization method that allowed us to attribute districts in older surveys to the appropriate state or UT as defined in 2021. This contrasts with the conventional approach to this problem of assigning averages since the harmonization method used in the study allows for the geographic granularity of current state geometry to be preserved and has been used previously to analyse prevalence in Zero‐Food over time (Subramanian et al., 2023). This choice was made to ensure that trends which could influence policy decisions are comparing the same regions over time.

### 2.5 Analysis

The mean prevalence of stunting, underweight and wasting was calculated for each survey year with 95% confidence intervals nationally and for each state and UT. Tables 1, 2, 3 present the consolidated data for the earliest and latest mean prevalence for each of these regions. Metrics for all years on stunting, underweight and wasting are presented in Supporting Information: Tables S1–S3, respectively. Boxplots were used to visualize the distribution of outcomes over time.

**Table 1:** Earliest and latest percent prevalence for moderate and severe stunting, grouped by earliest available survey year.

**Legend**	< 18	18‐22	22‐24	> 24
	Moderate		Severe	
	Earliest	2021	Earliest	2021
All India	20.5	16.0	22.6	16.3
**Earliest survey year: 1993**				
Arunachal Pradesh	14.7	11.8	29.5	13.3
Assam	23.5	16.1	22.4	18.7
Bihar	19.9	17.7	33.1	18.6
Goa	17.5	15.0	11.3	8.3
Gujarat	20.0	16.3	22.8	19.1
Haryana	25.0	14.1	17.0	10.8
Jharkhand	18.7	17.0	30.6	19.5
Karnataka	23.6	16.0	15.8	15.9
Kerala	16.1	15.7	9.5	10.1
Maharashtra	21.1	17.4	13.7	17.3
Manipur	11.7	13.1	11.7	9.3
Meghalaya	8.5	18.9	35.2	20.8
Mizoram	15.5	13.8	16.7	13.3
Nagaland	16.8	14.2	11.2	15.6
Odisha	20.6	17.2	22.7	14.9
Punjab	22.5	13.9	16.1	11.4
Rajasthan	16.9	14.8	25.0	15.1
Tripura	21.2	13.4	21.9	21.6
Uttar Pradesh	22.6	16.7	31.8	17.7
Uttarakhand	23.5	13.2	34.6	12.8
Jammu & Kashmir^a^	19.0	11.5	17.8	16.8
NCT of Delhi^a^	19.5	13.9	19.2	12.9
**Earliest survey year: 1999**				
Andhra Pradesh	23.3	16.0	15.6	11.3
Chhattisgarh	23.0	16.1	33.9	16.8
Himachal Pradesh	22.6	15.3	21.8	17.3
Madhya Pradesh	22.3	16.6	25.0	15.3
Sikkim	18.6	10.5	10.5	17.9
Tamil Nadu	17.7	15.6	13.5	12.1
Telangana	25.1	17.3	17.5	14.2
West Bengal	24.2	17.3	17.3	18.8
**Earliest survey year: 2006**				
Ladakh^a^	15.5	7.6	13.5	22.3
**Earliest survey year: 2016**				
Andaman & Nicobar Islands^a^	16.1	11.3	8.1	10.6
Chandigarh^a^	13.9	17.3	7.7	5.8
Dadra & Nagar Haveli and Daman & Diu^a^	15.6	20.1	20.2	13.9
Lakshadweep^a^	21.1	22.1	6.1	9.5
Puducherry^a^	13.5	12.6	10.3	16.7

^a^ Union Territories are marked

**Table 2:** Earliest and latest percent prevalence for moderate and severe underweight, grouped by earliest available survey year.

**Legend**	< 18	18‐22	22‐26	> 26
	Moderate		Severe	
	Earliest	2021	Earliest	2021
All India	23.4	17.5	18.2	10.9
**Earliest survey year: 1993**				
Andhra Pradesh	23.6	17.9	17.0	8.7
Arunachal Pradesh	18.6	7.0	10.3	5.3
Assam	24.9	18.2	13.3	12.9
Bihar	25.1	23.3	30.8	13.7
Chhattisgarh	28.2	19.4	29.8	12.8
Goa	16.5	14.0	6.9	6.6
Gujarat	23.7	20.7	17.1	14.6
Haryana	20.1	13.2	7.9	6.5
Himachal Pradesh	22.0	14.4	11.1	7.8
Jharkhand	21.6	22.6	17.9	16.0
Karnataka	27.9	19.0	16.4	10.0
Kerala	13.5	15.1	5.0	6.0
Madhya Pradesh	26.0	19.4	29.0	12.3
Maharashtra	25.5	20.3	18.3	12.4
Manipur	10.4	7.9	4.8	2.5
Meghalaya	18.3	15.3	12.7	8.3
Mizoram	9.8	8.0	4.6	3.8
Nagaland	12.5	14.4	3.5	7.9
Odisha	26.6	19.1	19.2	10.3
Punjab	23.2	12.8	12.9	5.7
Rajasthan	22.4	16.4	18.8	11.4
Sikkim	9.7	4.3	3.1	7.7
Tamil Nadu	25.9	13.6	14.7	7.4
Telangana	20.1	18.8	16.2	8.9
Tripura	19.7	14.5	18.3	9.2
Uttar Pradesh	26.7	17.2	26.5	12.2
Uttarakhand	23.3	11.1	17.9	7.9
West Bengal	29.7	19.0	22.7	10.2
Jammu & Kashmir^a^	16.9	12.8	12.1	10.9
NCT of Delhi^a^	21.2	14.7	11.8	6.7
**Earliest survey year: 1999**				
Ladakh^a^	13.0	17.2	8.9	13.3
**Earliest survey year: 2016**				
Andaman & Nicobar Islands^a^	12.3	13.7	3.8	10.6
Chandigarh^a^	15.4	13.2	6.2	7.6
Dadra & Nagar Haveli and Daman & Diu^a^	20.2	20.6	8.2	13.1
Lakshadweep^a^	18.4	18.6	5.3	13.4
Puducherry^a^	13.2	14.4	4.7	4.7

^a^ Union Territories are marked.

**Table 3:** Earliest and latest percent prevalence for moderate and severe wasting, grouped by earliest available survey year.

Wasting	< 9	9‐12	12‐15	> 15
	Moderate		Severe	
	Earliest	2021	Earliest	2021
All India	15.0	12.3	8.4	9.4
**Earliest survey year: 1993**				
Arunachal Pradesh	12.2	6.3	8.3	6.9
Assam	11.4	12.3	3.1	10.7
Bihar	20.8	16.5	13.9	12.0
Goa	12.3	15.5	5.2	10.1
Gujarat	16.1	14.9	11.0	13.0
Haryana	6.0	9.0	2.9	6.3
Jharkhand	11.9	16.3	3.0	11.5
Karnataka	20.3	12.9	8.5	9.6
Kerala	10.7	11.5	2.9	6.9
Maharashtra	19.1	14.9	13.5	12.2
Manipur	6.1	5.9	4.4	5.2
Meghalaya	12.0	9.1	8.5	6.4
Mizoram	5.2	5.1	1.2	5.6
Nagaland	6.9	11.1	3.5	6.6
Odisha	19.2	13.2	10.4	8.8
Punjab	15.4	9.0	6.0	5.7
Rajasthan	13.0	11.0	12.8	9.2
Tripura	16.8	8.9	5.8	8.1
Uttar Pradesh	17.9	11.9	11.1	9.9
Uttarakhand	13.0	9.2	6.4	7.1
Jammu & Kashmir^a^	11.7	11.1	6.0	11.6
NCT of Delhi^a^	12.9	8.0	4.4	7.3
**Earliest survey year: 1999**				
Andhra Pradesh	8.6	11.4	3.9	7.9
Chhattisgarh	19.5	13.2	6.9	10.7
Himachal Pradesh	11.3	8.6	5.0	6.9
Madhya Pradesh	16.8	14.0	11.7	8.6
Sikkim	4.3	2.7	3.1	10.3
Tamil Nadu	17.2	9.3	8.2	7.2
Telangana	6.4	13.4	3.5	9.8
West Bengal	13.9	12.2	5.5	6.9
**Earliest survey year: 2006**				
Ladakh^a^	14.3	6.5	6.6	12.9
**Earliest survey year: 2016**				
Andaman & Nicobar Islands^a^	8.1	14.4	5.8	4.8
Chandigarh^a^	12.3	7.6	10.8	5.7
Dadra & Nagar Haveli and Daman & Diu^a^	13.2	18.3	8.6	4.2
Lakshadweep^a^	11.4	8.8	3.5	13.2
Puducherry^a^	9.7	8.8	10.3	6.9

^a^ Union Territories are marked.

Standardized absolute change (SAC) was calculated for each outcome nationally and for each of the 36 states and UTs (Table 2). The SAC from year A to year B (where B > A) is defined as the total change in the outcome between year A and year B divided by the number of years elapsed between the two (B−A). This is calculated by the following formula, where p_A_ represents the outcome during year A and p_B_ represents the outcome in year B:

$$\begin{array}{ccc} \text{SAC} & = & \left(p_{B}-p_{A})/(B-A\right) \end{array}$$

A negative SAC indicates a decrease in the prevalence of an outcome (e.g., a decrease in stunting over time). The use of this metric allows for the comparison of prevalence change across varying time periods in units of percentage points per year (pp).

The R‐software (version 3.6.3) was used for data cleaning and metric computation. ArcGIS Pro and Microsoft Power BI were used for data visualization.

### 2.6 Role of funding

This study was supported by a grant from the Bill & Melinda Gates Foundation INV‐002992. The funders had no role in study design, data collection and analysis, decision to publish or preparation of the manuscript. All authors had access to the data. MN, OK and AK accessed and verified the sample survey data for the analysis.

### 2.7 Ethical statement

The data collection methods and survey content used by the NFHS are approved by the International Institute for Population Studies Institutional Review Board and the ICF Institutional Review Board. In each of the NFHS surveys, participants gave informed consent to their data being collected. The NFHS data are not collected specifically for this study and no one on the study team has access to identifiers linked to the data. These activities do not meet the regulatory definition of human subject research. As such, an Institutional Review Board (IRB) review is not required. The Harvard Longwood Campus IRB allows researchers to self‐determine when their research does not meet the requirements for IRB oversight via guidance online regarding when an IRB application is required using an IRB Decision Tool.

## 3 RESULTS

### 3.1 Patterns of change in stunting by severity

#### 3.1.1 Moderate stunting

**Figure 1:**
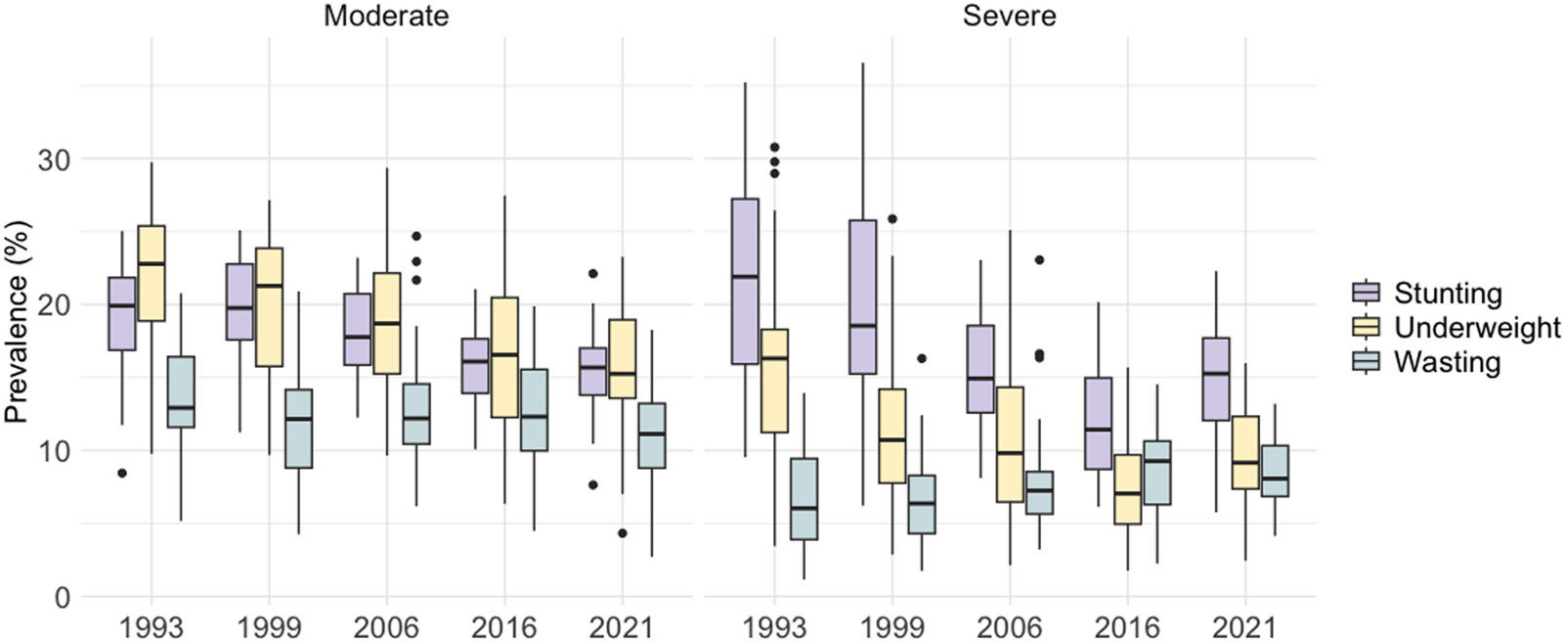
Summary distribution of prevalence for moderate and severe stunting, underweight and wasting across states and union territories (UTs) of India, 1993–2021.

Between 1993 and 2021, the nationwide prevalence of moderate stunting decreased from 20.5% (95% CI: 19.8%, 21.2%) in 1993 to 16.0% (95% CI: 15.8%, 16.3%) in 2021 (Table 1; Figure 1; Supporting Information: Table S1). The states with the greatest decrease in moderate stunting (not considering UTs since they had no earlier data before 2016) were Haryana at −0.39pp, Sikkim at −0.37pp and Uttarakhand at −0.37pp. Meghalaya, with an SAC of 0.37pp, saw the greatest increase in moderate stunting (Table 4).

**Table 4:** Standardized absolute change (SAC) for India and 36 states/union territories.

	Stunting				Underweight				Wasting			
	Severe		Moderate		Severe		Moderate		Severe		Moderate	
	Earliest‐Latest	2016–2021	Earliest‐Latest	2016–2021	Earliest‐Latest	2016–2021	Earliest‐Latest	2016–2021	Earliest‐Latest	2016–2021	Earliest‐Latest	2016–2021
All India	−0.23	0.26	−0.16	−0.32	−0.26	0.14	−0.21	−0.58	0.04	−0.04	−0.10	−0.48
Earliest survey year: 1993												
Andhra Pradesh	−0.20	0.28	−0.33	0.40	−0.30	0.50	−0.20	−0.62	0.18	−0.10	0.13	−0.74
Arunachal Pradesh	−0.58	−0.32	−0.10	−0.74	−0.18	−0.08	−0.41	−1.04	−0.05	−0.10	−0.21	−0.46
Assam	−0.13	1.04	−0.26	−0.54	−0.01	1.04	−0.24	0.32	0.27	0.84	0.03	0.24
Bihar	−0.52	0.00	−0.08	−0.68	−0.61	−0.06	−0.06	−0.50	−0.07	0.36	−0.15	0.02
Chhattisgarh	−0.78	−0.34	−0.31	−0.32	−0.61	−0.14	−0.31	−0.78	0.17	0.14	−0.29	−0.78
Goa	−0.11	0.24	−0.09	0.88	−0.01	0.32	−0.09	0.38	0.17	−0.10	0.11	0.98
Gujarat	−0.13	0.76	−0.14	−0.18	−0.09	0.56	−0.11	−0.56	0.07	−0.18	−0.04	−0.76
Haryana	−0.22	−0.84	−0.39	−0.38	−0.05	−0.30	−0.25	−1.12	0.12	−1.04	0.11	−0.72
Himachal Pradesh	−0.20	1.72	−0.33	0.64	−0.12	0.84	−0.27	0.10	0.09	0.12	−0.12	−0.46
Jharkhand	−0.40	0.30	−0.06	−0.58	−0.07	0.06	0.04	−0.98	0.30	−0.60	0.16	−0.72
Karnataka	0.01	−0.42	−0.27	−0.18	−0.23	−0.02	−0.32	−0.16	0.04	−0.22	−0.26	−0.54
Kerala	0.02	0.26	−0.01	0.48	0.04	0.16	0.06	0.60	0.15	−0.04	0.03	−0.12
Madhya Pradesh	−0.44	−0.28	−0.26	−0.26	−0.60	−0.34	−0.24	−1.18	−0.14	−0.84	−0.13	−1.08
Maharashtra	0.13	0.74	−0.13	0.10	−0.21	0.54	−0.19	0.12	−0.05	0.36	−0.15	−0.62
Manipur	−0.09	0.16	0.05	−0.68	−0.09	0.10	−0.09	0.12	0.03	0.60	−0.01	0.26
Meghalaya	−0.51	1.30	0.37	0.28	−0.16	0.38	−0.11	−0.32	−0.07	−0.66	−0.10	−0.18
Mizoram	−0.12	1.24	−0.06	−0.06	−0.03	0.40	−0.06	0.14	0.16	0.52	0.00	−0.04
Nagaland	0.16	1.24	−0.09	0.82	0.16	0.94	0.07	1.16	0.11	0.54	0.15	0.76
Odisha	−0.28	0.20	−0.12	−0.16	−0.32	0.18	−0.27	−0.74	−0.06	−0.10	−0.21	−0.70
Punjab	−0.17	0.40	−0.31	−0.32	−0.26	−0.20	−0.37	−0.32	−0.01	−0.12	−0.23	−0.64
Rajasthan	−0.35	−0.30	−0.07	−0.48	−0.26	0.00	−0.21	−1.00	−0.13	−0.34	−0.07	−0.98
Sikkim	0.34	1.30	−0.37	−0.66	0.21	0.98	−0.25	−0.40	0.33	0.94	−0.07	−1.28
Tamil Nadu	−0.06	−0.24	−0.10	−0.08	−0.26	0.14	−0.44	−0.52	−0.05	−0.60	−0.36	−0.64
Telangana	−0.15	1.62	−0.35	0.68	−0.26	0.28	−0.05	0.08	0.29	−0.04	0.32	−0.94
Tripura	−0.01	2.74	−0.28	0.66	−0.32	0.44	−0.19	0.00	0.08	−0.62	−0.28	−0.52
Uttar Pradesh	−0.50	0.08	−0.21	−0.66	−0.51	0.02	−0.34	−1.26	−0.04	0.16	−0.21	−0.68
Uttarakhand	−0.78	−0.36	−0.37	−0.76	−0.36	0.00	−0.44	−0.80	0.02	−0.80	−0.14	−0.48
West Bengal	0.07	1.48	−0.31	−0.08	−0.45	0.40	−0.38	−0.30	0.06	−0.36	−0.08	−0.38
Jammu & Kashmir^a^	−0.04	1.46	−0.27	−0.32	−0.04	1.32	−0.15	0.78	0.20	1.18	−0.02	0.58
NCT of Delhi^a^	−0.22	1.22	−0.20	−1.06	−0.18	−0.08	−0.23	−0.10	0.10	−0.30	−0.18	−1.22
Earliest survey year: 2006												
Ladakh^a^	0.59	2.18	−0.53	−1.34	0.30	1.84	0.28	1.64	0.42	1.84	−0.52	0.40
Earliest survey year: 2016												
Andaman & Nicobar Islands^a^	0.52	0.52	−0.96	−0.96	1.36	1.36	0.28	0.28	−0.18	−0.18	1.28	1.28
Chandigarh^a^	−0.38	−0.38	0.70	0.70	0.26	0.26	−0.44	−0.44	−1.02	−1.02	−0.96	−0.96
Dadra & Nagar Haveli and Daman & Diu^a^	−1.26	−1.26	0.90	0.90	0.98	0.98	0.08	0.08	−0.90	−0.90	1.02	1.02
Lakshadweep^a^	0.68	0.68	0.20	0.20	1.62	1.62	0.04	0.04	1.94	1.94	−0.52	−0.52
Puducherry^a^	1.28	1.28	−0.18	−0.18	0.00	0.00	0.24	0.24	−0.68	−0.68	−0.18	−0.18

*Note*: For Andhra Pradesh, Chhattisgarh, Himachal Pradesh, Madhya Pradesh, Sikkim, Tamil Nadu, Telangana and West Bengal, the earliest survey year is 1999 for stunting and wasting and 1993 for underweight. For Ladakh, the earliest for all metrics is 2006. For Andaman & Nicobar Islands, Chandigarh, Dadra & Nagar Haveli and Daman & Diu, Lakshadweep and Puducherry, the earliest for all metrics is 2016. For all other states and UTs, the earliest is 1993.

^a^ Union territories are marked.

#### 3.1.2 Severe stunting

From 1993 to 2021, the nationwide prevalence of severe stunting decreased from 22.6% (95% CI: 27.6%, 28.8%) in 1993 to 16.3% (95% CI: 13.8%, 14.1%) in 2021 (Table 1; Figure 1; Supporting Information: Table S1). The states with the greatest decrease in the prevalence of severe stunting (not considering UTs since they had no data before 2016) were Chhattisgarh with an SAC of −0.78pp and Uttarakhand with an SAC of −0.78pp. The state with the highest SAC (highest average annual increase) for severe stunting was Sikkim at 0.34pp (Table 4). While severe stunting has on the whole decreased over the past three decades, the 2016–2021 time range has seen an increase in severe stunting for several regions. There was an annual average increase of >1.00pp in severe stunting between 2016 and 2021 in Assam, Himachal Pradesh, Meghalaya, Mizoram, Nagaland, Sikkim, Telangana, Tripura, West Bengal, Jammu & Kashmir, Ladakh, NCT of Delhi and Puducherry (Table 4).

### 3.2 Patterns of change in underweight by severity

#### 3.2.1 Moderate underweight

The nationwide prevalence of moderate underweight decreased from 23.4% (95% CI: 22.8%, 24.0%) in 1993 to 17.5% (95% CI: 17.3%, 17.8%) in 2021 (Table 2; Figure 1; Supporting Information: Table S2). The states with the greatest decrease in moderate underweight were Tamil Nadu at −0.44pp and Uttarakhand at −0.44pp. Nagaland had an SAC of 0.07pp, indicating an increase in prevalence (Table 4). Comparing across outcomes in the 2016–2021 period, moderate underweight has seen the lowest SACs (greatest decrease in prevalence) since 2016, with Arunachal Pradesh, Haryana, Madhya Pradesh, Rajasthan and Uttar Pradesh all having an SAC of <‐1.00pp between 2016 and 2021 (Table 4).

#### 3.2.2 Severe underweight

The nationwide prevalence of severe underweight decreased from 18.2% (95% CI: 17.6%, 18.7%) in 1993 to 10.9% (95% CI: 10.7%, 11.1%) in 2021 (Table 2; Figure 1; Supporting Information: Table S2). The states with the greatest reduction in the prevalence of severe underweight were Chhattisgarh at −0.61pp, Bihar at −0.61pp and Madhya Pradesh at −0.60pp. Sikkim had the highest SAC (greatest increase in prevalence) for severe underweight at 0.21pp. Barring Kerala, Nagaland and Sikkim, all states have seen an annual average decrease in severe underweight since 1993. However, during the 2016–2021 period, we saw annual average increases in severe underweight in over half the regions, with Assam, Nagaland, Sikkim, Andaman & Nicobar Islands, Dadra & Nagar Haveli and Daman & Diu, Ladakh and Lakshadweep having SACs >0.9pp (Table 4).

### 3.3 Patterns of change in wasting by severity

#### 3.3.1 Moderate wasting

Nationwide prevalence of moderate wasting decreased from 15.0% (95% CI: 14.4%, 15.6%) in 1993 to 12.3% (95% CI: 12.0%, 12.5%) in 2021 (Table 3; Figure 1; Supporting Information: Table S3). The states with the greatest decrease in the prevalence of moderate wasting were Tamil Nadu at −0.36pp, Chhattisgarh at −0.29pp and Tripura at −0.28pp. Telangana saw the greatest increase in the prevalence of moderate wasting with an SAC of 0.32pp (Table 4). There have also been lower SACs (greater reduction) for moderate wasting when considering the 2016–2021 period as compared to the total 1993–2021 period. For example, Madhya Pradesh, Rajasthan, Sikkim and Telangana have all seen an SAC of <−0.9pp in the 2016–2021 period, though their cumulative SACs from the earliest–latest years are all >0.2pp, demonstrating an increase and then reduction in moderate wasting since 1993 (Table 4).

#### 3.3.2 Severe wasting

Nationwide prevalence of severe wasting increased from 8.4% (95% CI: 8.0%, 8.9%) in 1993 to 9.4% (95% CI: 9.2%, 9.6%) in 2021 (Table 3; Figure 1; Supporting Information: Table S3). Mirroring the all‐India trend, a majority of states have seen a slight increase in severe wasting since 1993. States with the greatest decrease in the prevalence of severe wasting were Madhya Pradesh at −0.14pp and Rajasthan at −0.13pp. The states with the highest SACs for severe wasting (greatest increase in prevalence) were Assam at 0.27pp, Jharkhand at 0.30pp, Sikkim at 0.33pp and Telangana at 0.29pp (Table 4). In the 2016–2021 time range, most states have seen a decrease in severe wasting. The state with the greatest increase in severe wasting in the 2016–2021 time range was Sikkim with an SAC of 0.94pp (Table 4).

## 4 DISCUSSION

These results contain three key findings. First, the study findings reveal a significantly greater reduction in severe stunting and underweight than moderate since 1993. On the other hand, the prevalence of severe wasting shows a slight increase since 1993, while moderate wasting has decreased. Third, though most indicators of CAF are noted to have decreased since 1993 (except for severe wasting), there has been significant heterogeneity in SAC, with certain states noted to have a high reduction and other states noted to have an increase in CAF. With varying regional levels of resources and implementation processes, interregional differences in the implementation of policy and management capacity can lead to wide differences in health outcomes. We are not aware of any study that has analysed anthropometric outcomes for 0–24 months segmented by severity for India, its states and its union territories using the most recent data. Therefore, these estimates can help states implement more tailored nutrition and health interventions, taking into account the severity of anthropometric failure (Ray, 2011).

Over the past few decades, India has launched national programs to address the direct and underlying determinants of child undernutrition. The most recent effort was the 2017 launch of a nutritional program referred to as POSHAN Abhiyaan, which aimed to address malnutrition by targeting the reduction of stunting, undernutrition, anaemia and low birth weight in children (Ministry of Women and Child Development, 2019). Analysis of data reveals the ongoing programs appear to have been most effective in reducing severe and moderate stunting and underweight, while the impact on severe wasting has remained a concern. Since stunting reflects chronic undernutrition, while wasting reflects acute undernutrition to a greater extent, the findings suggest that governmental interventions have been more effective for the former (Kerac et al., 2020).

An examination of the SAC of states and reduction of moderate child malnutrition rates could be attributed to higher investment by state governments in programs to combat undernutrition. For example, Tamil Nadu (SAC of −0.44pp since 1993 for moderate underweight) has the longstanding state‐led Tamil Nadu Nutritional Program targeting malnutrition in several thousand villages (Heaver, 2002). During the pandemic, Rajasthan (SAC of −1.00pp of moderate underweight in the 2016–2021 time period) launched the Indira Rasoi Yojana program in 2020 to distribute and subsidize fresh and nutritious food to target malnutrition (Sarva et al., 2019). While these state interventions may have had an impact on CAF reduction, other factors such as shifts in economic and demographic makeup of states could also have played a role (Sen & Mondal, 2012).

When examining the states that have seen the highest SACs in severe child malnutrition since 1993, the eight states deemed as ‘Empowered Action Group’ (EAG) states (Assam, Bihar, Chhattisgarh, Jharkhand, Madhya Pradesh, Odisha, Rajasthan, Uttar Pradesh and Uttarakhand) are noted to have a higher SAC (greater reduction in prevalence) for severe stunting and underweight. The findings indicate that possibly these states may have improved their outcomes over the years with the special resources provided by the Ministry of Health. These EAG states also have stricter monitoring through policies such as the federal Janani Surakhsa Yojana scheme that provides cash assistance to pregnant women. The National Rural Health Mission also targeted the health of the rural population, having results like an increased number of public sector institutional deliveries from 2004 to 2014 (National Rural Health Mission Annual Report, 2012; Bhavan, 2005; Dhingra & Dutta, 2011; Joe et al., 2018). The problem of moderate undernutrition in the EAG states has, however, not correspondingly decreased. Jharkhand, for example, has seen a 0.40pp reduction in severe stunting but little reduction (−0.06pp) in moderate stunting since 1993. These results could suggest these national programs focusing on EAGs have been more effective at higher severities than moderate severities. The key reasons contributing to such a situation, however, need to be analysed further.

In India, stunting SACs since 1993 were noted to be −0.16 for moderate and −0.23 for severe, both indicating a reduction in prevalence. Previous studies have compared India to other countries on anthropometric outcomes (Mejía‐Guevara et al., 2018). For example, in an analysis of stunting in children aged 6–23 months from 1991 to 2014, Bangladesh's stunting annual average reduction (AAR) was found to be 2.9 percentage points per year (pp), which was 2.23 times higher than India's stunting AAR of 1.3pp. Pakistan's stunting AAR (0.6pp) was 2.17 times lower than India's (Krishna et al., 2018). However, these results show significant interregional variation in outcomes across states in India. For example, Uttarakhand SAC for severe stunting (0.78pp) was 3.39 times higher than the national (0.23pp) and its AAR for moderate stunting (0.37pp) was 2.31 times higher than the national (0.16pp)—more comparable to Bangladesh's rate of reduction. Mizoram's SAC for severe stunting (0.12pp) was 1.92 times lower than the national (0.23pp) and its SAC for moderate stunting (0.06pp) was 2.67 times lower than the national (0.16pp)—comparable to Pakistan's.

These findings should be contextualized with some limitations. First, the 1993 survey omitted child height or weight for many states, leading to a sparser sample population in that year. Second, older surveys had incorrect ages, which led to wider tails in the height‐for‐age and weight‐for‐age distributions, a change that could cause higher apparent stunting and underweight prevalences, especially in the severe categories (Pullum & Staveteig, 2017). Therefore, since the earlier estimates may be exaggerated to a greater extent, the observed reduction in stunting and underweight over time may be, in part, a reflection of improved age measurements. Third, NFHS‐5 was disrupted due to COVID‐19; commenced in 2019, it paused and resumed in late 2020–21. Studies have shown that some anthropometric indicators were affected by the pandemic, such as the prevalence of underweight seeing a 1.87% increase from 2019 to 2021 (Ko et al., 2023). Further studies should investigate any influence of the pandemic on severe and moderate anthropometric failure, both positive influence due to newly created governmental health interventions and negative influence due to economic crisis and unemployment (Ghosh et al., 2020). Fourth, the wasting can vary from season to season and surveys were conducted in different regions in different seasons (Dwivedi et al., 2023). NFHS is still considered to be a reliable, good‐quality data source for analyses on child anthropometric outcomes (Assaf et al., 2015). Fifth, due to the variability in state populations, some estimates are calculated on smaller sample sizes (Supporting Information: Tables S5–S7).

Reduction in the prevalence of underweight and stunting suggests that governmental interventions since 1993 have broadly resulted in better nutrition for India's children. However, this paper shows some regions still lagging behind in anthropometric outcomes especially when segmenting by severity, suggesting that policymakers should target those regions with interventions that improve access to nutritious food. State‐ and regional‐level NFHS data have often been helpful in informing regional policy, from finding regional disparities in under‐five mortality to addressing micronutrient deficiency variation across states (Bora, 2020; Sharma et al., 2020). Improving children's nutrition can then reduce the prevalence of anthropometric failure. Access to food, however, is not the only determinant. In addition to undernutrition, anthropometric outcomes are also impacted by infections. Undernourished children are more likely to suffer undernutrition, and infections can cause undernutrition through malabsorption, reduced appetite and energy being diverted from growth to fight off infections, as observed in one study of children in Tehran (Nematian et al., 2008). Parental nutritional status and household socioeconomic conditions were also shown to be correlated with anthropometric failure in stunting, underweight and wasting in a study of 35 low‐ and middle‐income countries (Li et al., 2020). Targeting not only undernutrition but also infections and socioeconomic well‐being of families can help improve these outcomes.

## AUTHOR CONTRIBUTIONS

*Conceptualization and Design*: S. V. Subramanian, Rockli Kim. *Data Acquisition and Analysis*: Menaka Narayanan, Omar Karlsson, Akhil Kumar. *Data Interpretation*: Menaka Narayanan, Omar Karlsson, Akhil Kumar, Thomas W. Pullum, Rockli Kim, S. V. Subramanian. *Writing of the Manuscript*: Menaka Narayanan, Omar Karlsson. *Critical Revisions*: Omar Karlsson, Akhil Kumar, Thomas W. Pullum, S. V. Subramanian, Rockli Kim. *Overall Supervision*: Rockli Kim, S. V. Subramanian.

## CONFLICT OF INTEREST STATEMENT

The authors declare no conflict of interest.

## Supporting information

Supporting information.

## Data Availability

The study is based on publicly available data and can be accessed from https://dhsprogram.com/data/available-datasets.cfm.

## References

[mcn13751-bib-0001] Assaf, S. , Kothari, M. T. , & Pullum, T. W. (2015). An Assessment of the Quality of DHS Anthropometric Data (pp. 2005–2014). ICF International.

[mcn13751-bib-0002] Balarajan, Y. , & Reich, M. R. (2016). Political economy of child nutrition policy: A qualitative study of India's integrated child development services (ICDS) scheme. Food Policy, 62, 88–98. 10.1016/j.foodpol.2016.05.001

[mcn13751-bib-0003] Baru, R. , Acharya, A. , Acharya, S. , Kumar, A. S. , & Nagaraj, K. (2010). Inequities in access to health services in India: Caste, class and region. Economic and Political Weekly, 45(38), 49–58. http://www.jstor.org/stable/25742094

[mcn13751-bib-0043] Bhat, P. M. , & Zavier, F. (1999). Findings of national family health survey: Regional analysis. Economic and Political Weekly, 3008–3032.

[mcn13751-bib-0004] Bhavan, N. (2005). JANANI SURAKSHA YOJANA. https://nhm.gov.in/WriteReadData/l892s/97827133331523438951.pdf

[mcn13751-bib-0005] Bora, J. K. (2020). Factors explaining regional variation in under‐five mortality in India: An evidence from NFHS‐4. Health & Place, 64, 102363. 32838888 10.1016/j.healthplace.2020.102363

[mcn13751-bib-0007] Chandrakant, S. (2008). Child in India. Indian Journal of Psychiatry, 50(2), 85. 19742226 10.4103/0019-5545.42393 PMC2738347

[mcn13751-bib-0044] Dhingra, B. , & Dutta, A. K. (2011). National rural health mission. The Indian Journal of Pediatrics, 78, 1520–1526. 21830029 10.1007/s12098-011-0536-4

[mcn13751-bib-0008] Dwivedi, L. K. , Bhatia, M. , Bansal, A. , Mishra, R. , P, S. , Jana, S. , Subramanian, S. V. , & Unisa, S. (2023). Role of seasonality variation in prevalence and trend of childhood wasting in India: An empirical analysis using National Family Health Surveys, 2005–2021. Health Science Reports, 6(2), e1093. https://onlinelibrary.wiley.com/doi/full/10.1002/hsr2.1093 36817627 10.1002/hsr2.1093 PMC9935817

[mcn13751-bib-0009] Ganguly, S. , & Unisa, S. (2010). Trends of infertility and childlessness in India: Findings from NFHS data. Facts, Views & Vision in ObGyn, 2(2), 131–138. PMC4188020 25300753

[mcn13751-bib-0010] Ghosh, A. , Nundy, S. , & Mallick, T. K. (2020). How India is dealing with COVID‐19 pandemic. Sensors International, 1, 100021. 10.1016/j.sintl.2020.100021 34766039 PMC7376361

[mcn13751-bib-0012] Heaver, R. (2002). India's Tamil Nadu Nutrition Program: lessons and issues in management and capacity development. https://www.unscn.org/web/archives_resources/files/Indias_Tamil_Nadu_Nutrition_Program.pdf

[mcn13751-bib-0013] Heemann, M. , Kim, R. , Vollmer, S. , & Subramanian, S. V. (2021). Assessment of undernutrition among children in 55 low‐and middle‐income countries using dietary and anthropometric measures. JAMA Network Open, 4(8), e2120627. https://jamanetwork.com/journals/jamanetworkopen/fullarticle/2782986 34383059 10.1001/jamanetworkopen.2021.20627 PMC12549096

[mcn13751-bib-0014] Hemalatha, R. , Pandey, A. , Kinyoki, D. , Ramji, S. , Lodha, R. , Kumar, G. A. , Kassebaum, N. J. , Borghi, E. , Agrawal, D. , Gupta, S. S. , Laxmaiah, A. , Kar, A. , Mathai, M. , Varghese, C. M. , Awasthi, S. , Bansal, P. G. , Chakma, J. K. , Collison, M. , Dwivedi, S. , … Dandona, L. (2020). Mapping of variations in child stunting, wasting and underweight within the states of India: The Global Burden of Disease Study 2000–2017. EClinicalMedicine, 22, 100317. 32510044 10.1016/j.eclinm.2020.100317 PMC7264980

[mcn13751-bib-0015] ICF International . (2012). MEASURE DHS Biomarker Field Manual. ICF International.

[mcn13751-bib-0018] Joe, W. , Perkins, J. M. , Kumar, S. , Rajpal, S. , & Subramanian, S. V. (2018). Institutional delivery in India, 2004–14: Unravelling the equity‐enhancing contributions of the public sector. Health Policy and Planning, 33(5), 645–653. 29659831 10.1093/heapol/czy029

[mcn13751-bib-0019] Jose, S. , & Hari, K. S. (2015). Progress in reducing child under‐nutrition: Evidence from Maharashtra. Economic and Political Weekly, 50(3), 23–26. http://www.jstor.org/stable/24481117

[mcn13751-bib-0020] Karlsson, O. , Kim, R. , Sarwal, R. , James, K. S. , & Subramanian, S. V. (2021). Trends in underweight, stunting, and wasting prevalence and inequality among children under three in Indian states, 1993–2016. Scientific Reports, 11(1), 14137. https://www.ncbi.nlm.nih.gov/pmc/articles/PMC8266817/ 34238988 10.1038/s41598-021-93493-1 PMC8266817

[mcn13751-bib-0021] Kerac, M. , McGrath, M. , Connell, N. , Kompala, C. , Moore, W. H. , Bailey, J. , Bandsma, R. , Berkley, J. A. , Briend, A. , Collins, S. , Girma, T. , & Wells, J. C. (2020). ‘Severe malnutrition’: Thinking deeply, communicating simply. BMJ Global Health, 5(11), e003023. https://www.ncbi.nlm.nih.gov/pmc/articles/PMC7677332/ 10.1136/bmjgh-2020-003023 PMC7677332 33208313

[mcn13751-bib-0022] Ko, S. , Kim, R. , & Subramanian, S. V. (2023). Patterns in child health outcomes before and after the COVID‐19 outbreak in India. JAMA Network Open, 6(6), e2317055. 37273207 10.1001/jamanetworkopen.2023.17055 PMC10242422

[mcn13751-bib-0023] Krishna, A. , Mejía‐Guevara, I. , McGovern, M. , Aguayo, V. M. , & Subramanian, S. V. (2018). Trends in inequalities in child stunting in South Asia. Maternal & child nutrition, 14(Suppl. 4), e12517. https://onlinelibrary.wiley.com/doi/full/10.1111/mcn.12517 29048726 10.1111/mcn.12517 PMC6519254

[mcn13751-bib-0025] Li, Z. , Kim, R. , Vollmer, S. , & Subramanian, S. V. (2020). Factors associated with child stunting, wasting, and underweight in 35 low‐and middle‐income countries. JAMA network open, 3(4), e203386. https://jamanetwork.com/journals/jamanetworkopen/article-abstract/2764662 32320037 10.1001/jamanetworkopen.2020.3386 PMC7177203

[mcn13751-bib-0026] Martorell, R. (1999). The nature of child malnutrition and its long‐term implications. Food and Nutrition Bulletin, 20(3), 288–292. 10.1177/156482659902000304

[mcn13751-bib-0028] Mejía‐Guevara, I. , Corsi, D. J. , Perkins, J. M. , Kim, R. , & Subramanian, S. V. (2018). Variation in anthropometric status and growth failure in low‐and middle‐income countries. Pediatrics, 141(3), e20172183. 10.1542/peds.2017-2183 29472493

[mcn13751-bib-0029] Ministry of Women and Child Development (2019). POSHAN Abhiyaan (National Nutrition Mission). Government of India. https://icds-wcd.nic.in/nnm/home.html

[mcn13751-bib-0030] National Rural Health Mission Home . Government of India. https://nhm.gov.in/index1.php

[mcn13751-bib-0031] National Rural Health Mission Annual Report . (2012−13). Government of India. https://main.mohfw.gov.in/sites/default/files/CHAPTER%202.pdf

[mcn13751-bib-0032] Nematian, J. , Gholamrezanezhad, A. , & Nematian, E. (2008). Giardiasis and other intestinal parasitic infections in relation to anthropometric indicators of malnutrition: a large, population‐based survey of schoolchildren in Tehran. Annals of Tropical Medicine and Parasitology, 102(3), 209–214. 18348775 10.1179/136485908X267876

[mcn13751-bib-0033] Pullum, T. W. , & Staveteig, S. (2017). An assessment of the quality and consistency of age and date reporting in DHS surveys, 2000–2015. ICF. http://dhsprogram.com/pubs/pdf/MR19/MR19.pdf

[mcn13751-bib-0034] Ray, S. (2011). Evidence‐based preventive interventions for targeting under‐nutrition in the Indian context. Indian Journal of Public Health, 55(1), 1–6. 21727673 10.4103/0019-557X.82531

[mcn13751-bib-0045] Sarva, M. , Sidana, A. , Singh, R. R. , & Mandala, G. N. (2019). Revisiting state subsidy food programme model for India. International Journal of Recent Technology and Engineering, 8(4), 2545–2548.

[mcn13751-bib-0035] Sen, J. , & Mondal, N. (2012). Socio‐economic and demographic factors affecting the composite index of anthropometric failure (CIAF). Annals of Human Biology, 39(2), 129–136. 22324839 10.3109/03014460.2012.655777

[mcn13751-bib-0036] Sharma, H. , Singh, S. K. , & Srivastava, S. (2020). Socio‐economic inequality and spatial heterogeneity in anaemia among children in India: Evidence from NFHS‐4 (2015–16). Clinical Epidemiology and Global Health, 8(4), 1158–1171.

[mcn13751-bib-0037] Subramanian, S. V. , Ambade, M. , Sharma, S. , Kumar, A. , & Kim, R. (2023). Prevalence of Zero‐Food among infants and young children in India: Patterns of change across the states and union territories of India, 1993–2021. EClinicalMedicine, 58, 101890. https://www.thelancet.com/journals/eclinm/article/PIIS2589-5370(23)00067-6/fulltext 37065175 10.1016/j.eclinm.2023.101890 PMC10102207

[mcn13751-bib-0038] United Nations Development Program . (2022). The SDGs in action. https://www.undp.org/sustainable-development-goals

[mcn13751-bib-0039] World Health Organization . (2006). WHO Child Growth Standards: Length/Height for Age, Weight‐for‐Age, Weight‐for‐Length, Weight‐for‐Height and Body Mass Index‐for‐Age, Methods and Development.

[mcn13751-bib-0040] World Health Organization . (2019). Nutrition Landscape Information System (NLiS) country profile indicators: Interpretation guide, second edition. https://apps.who.int/iris/bitstream/handle/10665/332223/9789241516952-eng.pdf

