# Supplementary material for: Prevalence of severe and moderate anthropometric failure among children in India, 1993–2021
Source: Matern Child Nutr. 2024 Dec 4;21(2):e13751. doi: 10.1111/mcn.13751 (PMC11956042; doi:10.1111/mcn.13751)
Supplement: Supplementary file 1 — Supporting information. [file MCN-21-e13751-s001.pdf]

Supplementary Content

Table S1. Prevalence of Stunting (%) and 95% Confidence Intervals for the States/UTs of India, 1993-2021
Table S2. Prevalence of Underweight (%) and 95% Confidence Intervals for the States/UTs of India, 1993-2021
Table S3. Prevalence of Wasting (%) and 95% Confidence Intervals for the States/UTs of India, 1993-2021
Table S4. Analytical Samples for Stunting, Underweight, and Wasting
Table S5. Analytical Samples by State/Union Territory for Stunting
Table S6. Analytical Samples by State/Union Territory for Underweight
Table S7. Analytical Samples by State/Union Territory for Wasting

This supplementary material has been provided by the authors to give readers additional information about their work

Table S1. Prevalence of Stunting (%) and 95% Confidence Intervals for the States/UTs of India, 1993-2021

	1993		1999		2006		2016		2021	
	Moderate	Severe	Moderate	Severe	Moderate	Severe	Moderate	Severe	Moderate	Severe
All India	20.49 [19.82-21.17]	22.58 [21.89-23.29]	20.75 [20.14-21.37]	21.53 [20.92-22.16]	19.04 [18.45-19.65]	16.4 [15.84-16.97]	17.64 [17.4-17.89]	14.96 [14.73-15.2]	16 [15.75-16.25]	16.25 [16-16.51]
**States**										
Andhra Pradesh			23.33 [19.25-27.98]	15.56 [12.17-19.68]	17.65 [14.96-20.7]	10.88 [8.75-13.45]	13.99 [12.04-16.2]	9.89 [8.24-11.82]	15.97 [13.84-18.37]	11.31 [9.5-13.42]
Arunachal Pradesh	14.74 [10-21.21]	29.49 [22.86-37.11]	12.96 [9.1-18.14]	15.74 [11.47-21.22]	16.49 [12.58-21.31]	14.34 [10.69-18.96]	15.51 [13.67-17.55]	14.91 [13.11-16.92]	11.76 [10.34-13.34]	13.27 [11.77-14.92]
Assam	23.51 [20.38-26.96]	22.41 [19.34-25.81]	15.25 [12.1-19.05]	36.56 [32.05-41.32]	19.57 [16.23-23.41]	17.23 [14.08-20.92]	18.84 [17.55-20.19]	13.53 [12.41-14.72]	16.13 [14.97-17.37]	18.7 [17.46-20]
Bihar	19.91 [17.72-22.3]	33.08 [30.44-35.83]	17.43 [15.31-19.76]	31.72 [29.06-34.51]	21.96 [19.3-24.87]	18.65 [16.17-21.42]	21 [20.17-21.85]	18.53 [17.74-19.34]	17.65 [16.8-18.53]	18.55 [17.68-19.45]
Chhattisgarh			22.99 [17.33-29.82]	33.91 [27.26-41.25]	23.02 [19.75-26.66]	22.14 [18.92-25.74]	17.74 [16.51-19.04]	18.51 [17.26-19.83]	16.06 [14.77-17.43]	16.84 [15.53-18.23]
Goa	17.54 [14.39-21.21]	11.27 [8.74-14.43]	11.48 [7.82-16.56]	6.22 [3.65-10.42]	12.25 [9.01-16.45]	10.26 [7.31-14.23]	10.64 [6.52-16.9]	7.09 [3.86-12.68]	15.04 [9.91-22.16]	8.27 [4.64-14.32]
Gujarat	20.03 [17.32-23.04]	22.81 [19.96-25.94]	20.5 [17.64-23.69]	22.25 [19.29-25.53]	22.06 [18.74-25.77]	21.12 [17.87-24.79]	17.14 [15.71-18.67]	15.26 [13.91-16.72]	16.25 [15.06-17.51]	19.07 [17.8-20.4]
Haryana	25.03 [22.09-28.23]	17.04 [14.53-19.87]	21.35 [18.2-24.89]	26.56 [23.11-30.32]	22.66 [19.06-26.71]	18.52 [15.22-22.34]	15.98 [14.66-17.39]	14.97 [13.69-16.35]	14.09 [12.75-15.54]	10.79 [9.6-12.09]
Himachal Pradesh			22.55 [19.22-26.27]	21.81 [18.53-25.49]	15.18 [11.73-19.42]	12.8 [9.63-16.81]	12.1 [10.25-14.23]	8.71 [7.14-10.59]	15.34 [13.24-17.7]	17.33 [15.11-19.8]
Jharkhand	18.66 [12.93-26.16]	30.6 [23.39-38.9]	18.89 [14.65-24]	27.04 [22.08-32.65]	19.58 [16.41-23.2]	21.48 [18.18-25.2]	19.9 [18.74-21.12]	17.96 [16.84-19.13]	17.01 [15.82-18.27]	19.47 [18.21-20.79]
Karnataka	23.56 [20.86-26.5]	15.75 [13.48-18.32]	17.27 [14.68-20.19]	15.88 [13.4-18.73]	16.87 [14-20.2]	20.43 [17.3-23.96]	16.91 [15.51-18.41]	17.96 [16.53-19.49]	15.98 [14.68-17.37]	15.91 [14.61-17.3]
Kerala	16.1 [13.56-19]	9.54 [7.58-11.95]	16.97 [13.55-21.03]	7.46 [5.23-10.52]	18.92 [15.25-23.23]	8.11 [5.73-11.36]	13.34 [11.2-15.83]	8.77 [7.03-10.9]	15.68 [13.47-18.16]	10.05 [8.28-12.16]
Madhya Pradesh			22.27 [20.06-24.65]	24.96 [22.65-27.42]	21.13 [18.76-23.71]	17.02 [14.86-19.42]	17.85 [17.06-18.68]	16.68 [15.9-17.48]	16.61 [15.68-17.6]	15.26 [14.35-16.21]
Maharashtra	21.09 [18.32-24.15]	13.66 [11.39-16.3]	24.35 [21.84-27.05]	13.9 [11.93-16.14]	23.2 [20.52-26.11]	12.91 [10.85-15.3]	16.86 [15.58-18.22]	13.62 [12.46-14.87]	17.39 [16.11-18.75]	17.26 [15.99-18.62]
Manipur	11.74 [8.17-16.58]	11.74 [8.17-16.58]	18.2 [14.77-22.23]	12.86 [9.96-16.46]	14.24 [11.77-17.14]	8.12 [6.25-10.47]	16.39 [14.9-18.01]	8.54 [7.44-9.79]	13.05 [11.2-15.15]	9.29 [7.73-11.14]
Meghalaya	8.45 [4.86-14.29]	35.21 [27.81-43.4]	11.24 [7.97-15.62]	29.96 [24.77-35.73]	16.46 [12.31-21.67]	23.05 [18.18-28.76]	17.53 [15.66-19.58]	14.28 [12.56-16.18]	18.91 [17.34-20.59]	20.84 [19.21-22.58]
Mizoram	15.52 [10.86-21.68]	16.67 [11.84-22.96]	19.44 [15.27-24.42]	14.58 [10.96-19.15]	15.06 [11.2-19.95]	13.13 [9.53-17.81]	14.13 [12.55-15.87]	7.09 [5.96-8.42]	13.79 [11.62-16.29]	13.32 [11.18-15.79]
Nagaland	16.81 [12.53-22.18]	11.21 [7.74-15.95]	19.76 [15.26-25.18]	14.92 [11-19.92]	14.62 [12.17-17.47]	11.26 [9.1-13.85]	10.08 [8.67-11.69]	9.36 [8-10.92]	14.16 [12.22-16.34]	15.61 [13.58-17.87]
Orissa	20.6 [17.88-23.62]	22.7 [19.87-25.81]	22.43 [19.75-25.37]	18.54 [16.06-21.3]	21.3 [18.28-24.68]	18.28 [15.45-21.5]	17.95 [16.76-19.21]	13.94 [12.87-15.08]	17.19 [15.91-18.57]	14.87 [13.66-16.17]
Punjab	22.51 [19.4-25.96]	16.08 [13.4-19.18]	19.79 [16.43-23.64]	18.09 [14.86-21.83]	16.59 [13.46-20.29]	15.5 [12.47-19.11]	15.47 [13.91-17.18]	9.43 [8.19-10.84]	13.91 [12.42-15.54]	11.41 [10.05-12.93]
Rajasthan	16.93 [14.52-19.65]	24.97 [22.13-28.05]	24.04 [21.95-26.26]	28.28 [26.06-30.6]	18.29 [15.67-21.25]	15.85 [13.39-18.67]	17.18 [16.25-18.15]	16.6 [15.68-17.56]	14.79 [13.85-15.79]	15.08 [14.13-16.08]
Sikkim			18.6 [14.31-23.82]	10.47 [7.27-14.83]	13.3 [9.15-18.94]	14.36 [10.04-20.13]	13.71 [10.65-17.47]	11.42 [8.64-14.96]	10.45 [6.91-15.49]	17.91 [13.2-23.83]
Tamil Nadu			17.72 [15.19-20.56]	13.48 [11.26-16.06]	15.97 [13.2-19.2]	11.28 [8.95-14.14]	15.97 [14.61-17.42]	13.25 [12.01-14.61]	15.64 [14.24-17.15]	12.05 [10.81-13.42]
Telangana			25.08 [20.6-30.16]	17.46 [13.66-22.06]	17.65 [14.96-20.7]	10.88 [8.75-13.45]	13.92 [11.77-16.4]	6.15 [4.73-7.96]	17.28 [15.86-18.79]	14.2 [12.9-15.6]
Tripura	21.17 [15.13-28.8]	21.9 [15.76-29.59]	16.22 [11.11-23.06]	21.62 [15.72-28.98]	17.43 [12.95-23.05]	11.93 [8.25-16.94]	10.13 [7.67-13.26]	7.93 [5.77-10.8]	13.37 [11.09-16.03]	21.56 [18.73-24.68]
Uttar Pradesh	22.59 [21.02-24.24]	31.8 [30.04-33.62]	24.2 [22.09-26.43]	28.49 [26.25-30.83]	20.6 [18.93-22.37]	22.21 [20.48-24.03]	20.01 [19.38-20.66]	17.27 [16.67-17.88]	16.69 [16.04-17.36]	17.7 [17.04-18.39]
Uttaranchal	23.53 [19.66-27.89]	34.56 [30.1-39.31]	24.56 [17.53-33.28]	21.93 [15.27-30.45]	18.9 [15.43-22.94]	16.75 [13.47-20.64]	16.98 [15.38-18.7]	14.64 [13.15-16.27]	13.15 [11.45-15.07]	12.78 [11.1-14.68]
West Bengal			24.22 [21.2-27.52]	17.28 [14.67-20.25]	21.24 [18.5-24.27]	13.86 [11.6-16.48]	17.65 [16.03-19.39]	11.4 [10.07-12.87]	17.28 [15.72-18.97]	18.78 [17.15-20.51]
**Union Territories (UTs)**										
Andaman & Nicobar Islands							16.09 [12.12-21.06]	8.05 [5.3-12.02]	11.26 [7.11-17.36]	10.6 [6.59-16.6]
Chandigarh							13.85 [7.36-24.52]	7.69 [3.24-17.18]	17.31 [9.26-30.04]	5.77 [1.87-16.42]
Dadra & Nagar Haveli and Daman & Diu							15.64 [11.59-20.76]	20.16 [15.59-25.68]	20.08 [15.52-25.58]	13.93 [10.13-18.87]
Jammu & Kashmir	19 [16.01-22.4]	17.79 [14.88-21.12]	19.07 [15.96-22.62]	17.76 [14.75-21.23]	15.48 [12.28-19.33]	13.51 [10.52-17.19]	12.96 [11.72-14.31]	9.53 [8.45-10.72]	11.45 [10.06-12.99]	16.81 [15.17-18.6]
Ladakh					15.48 [12.28-19.33]	13.51 [10.52-17.19]	14.29 [10.44-19.25]	11.43 [8.01-16.06]	7.64 [4.39-12.98]	22.29 [16.46-29.47]
Lakshadweep							21.05 [14.53-29.49]	6.14 [2.96-12.32]	22.11 [14.88-31.54]	9.47 [5-17.21]
NCT of Delhi	19.45 [16.8-22.41]	19.19 [16.55-22.13]	19.02 [15.42-23.23]	18.77 [15.19-22.96]	17.88 [13.79-22.88]	22.26 [17.73-27.57]	19.21 [15.84-23.1]	6.84 [4.85-9.57]	13.9 [11.83-16.27]	12.94 [10.94-15.25]
Puducherry							13.49 [10.26-17.54]	10.26 [7.46-13.96]	12.64 [9.17-17.17]	16.73 [12.73-21.67]

Table S2. Prevalence of Underweight (%) and 95% Confidence Intervals for the States/UTs of India, 1993-2021

	1993		1999		2006		2016		2021	
	Moderate	Severe	Moderate	Severe	Moderate	Severe	Moderate	Severe	Moderate	Severe
All India	23.42 [22.81-24.03]	18.15 [17.6-18.71]	21.68 [21.06-22.31]	14.33 [13.81-14.87]	20.31 [19.71-20.94]	11.99 [11.5-12.49]	20.38 [20.12-20.65]	10.17 [9.97-10.37]	17.52 [17.26-17.77]	10.92 [10.72-11.14]
**States**										
Andhra Pradesh	23.64 [20.1-27.58]	16.97 [13.91-20.54]	22.78 [18.74-27.4]	9.17 [6.59-12.61]	15.59 [13.05-18.51]	6.47 [4.85-8.58]	20.99 [18.65-23.53]	6.16 [4.87-7.76]	17.94 [15.72-20.41]	8.73 [7.15-10.61]
Arunachal Pradesh	18.59 [13.24-25.47]	10.26 [6.38-16.09]	13.43 [9.49-18.65]	7.87 [4.95-12.29]	18.28 [14.17-23.26]	7.89 [5.25-11.68]	12.16 [10.51-14.02]	5.67 [4.55-7.04]	7.01 [5.94-8.25]	5.25 [4.33-6.36]
Assam	24.92 [21.72-28.43]	13.32 [10.9-16.19]	22.03 [18.3-26.29]	13.8 [10.8-17.48]	23.62 [19.99-27.67]	9.79 [7.41-12.82]	16.55 [15.34-17.85]	7.71 [6.86-8.66]	18.18 [16.99-19.43]	12.86 [11.84-13.96]
Bihar	25.13 [22.73-27.69]	30.77 [28.19-33.47]	25.65 [23.17-28.29]	21.45 [19.14-23.95]	28.69 [25.74-31.83]	20.54 [17.96-23.4]	25.84 [24.95-26.76]	14.01 [13.31-14.74]	23.27 [22.34-24.22]	13.74 [12.99-14.52]
Chhattisgarh	28.24 [23.12-34]	29.77 [24.54-35.59]	24.14 [18.36-31.05]	25.86 [19.9-32.88]	27.59 [24.07-31.41]	16.17 [13.37-19.43]	23.32 [21.95-24.75]	13.48 [12.38-14.65]	19.39 [18.04-20.82]	12.8 [11.67-14.02]
Goa	16.49 [13.43-20.09]	6.89 [4.94-9.53]	14.35 [10.22-19.79]	2.87 [1.3-6.24]	15.56 [11.9-20.1]	4.3 [2.52-7.27]	12.06 [7.63-18.54]	4.96 [2.39-10.05]	13.97 [9.09-20.87]	6.62 [3.48-12.23]
Gujarat	23.74 [20.84-26.91]	17.11 [14.59-19.97]	23.13 [20.12-26.45]	14.06 [11.65-16.87]	23.36 [19.97-27.14]	14.39 [11.67-17.63]	23.53 [21.91-25.23]	11.79 [10.58-13.11]	20.72 [19.44-22.06]	14.58 [13.47-15.76]
Haryana	20.05 [17.36-23.04]	7.86 [6.15-10]	17.71 [14.8-21.04]	10.24 [8.02-13]	23.09 [19.46-27.17]	14.38 [11.46-17.9]	18.8 [17.38-20.29]	7.97 [7.02-9.04]	13.21 [11.93-14.6]	6.5 [5.6-7.54]
Himachal Pradesh	21.98 [19.02-25.25]	11.06 [8.91-13.65]	21.63 [18.36-25.3]	10.72 [8.38-13.62]	19.64 [15.74-24.24]	8.04 [5.57-11.46]	13.94 [11.96-16.19]	3.58 [2.61-4.9]	14.42 [12.42-16.69]	7.79 [6.31-9.58]
Jharkhand	21.64 [15.48-29.41]	17.91 [12.3-25.33]	24.44 [19.69-29.92]	23.33 [18.67-28.75]	24.33 [20.86-28.19]	24.33 [20.86-28.19]	27.46 [26.14-28.81]	15.69 [14.63-16.8]	22.61 [21.3-23.98]	15.98 [14.84-17.19]
Karnataka	27.93 [25.05-31.01]	16.44 [14.12-19.05]	20.17 [17.4-23.25]	13.26 [10.98-15.93]	19.54 [16.47-23.02]	10.48 [8.21-13.29]	19.67 [18.18-21.26]	10.07 [8.97-11.29]	18.95 [17.58-20.39]	9.98 [8.95-11.11]
Kerala	13.53 [11.2-16.27]	4.99 [3.6-6.87]	15.42 [12.17-19.36]	4.88 [3.14-7.53]	15.14 [11.83-19.16]	4.05 [2.46-6.61]	12.14 [10.09-14.54]	5.17 [3.85-6.9]	15.07 [12.95-17.48]	6.03 [4.69-7.72]
Madhya Pradesh	26.03 [23.71-28.49]	28.97 [26.56-31.51]	26.31 [23.95-28.81]	21.95 [19.75-24.32]	29.35 [26.67-32.18]	21.41 [19.03-24.01]	25.33 [24.42-26.26]	14.01 [13.29-14.76]	19.39 [18.41-20.41]	12.33 [11.52-13.19]
Maharashtra	25.46 [22.48-28.7]	18.3 [15.7-21.23]	25.31 [22.76-28.04]	13.14 [11.22-15.32]	19.66 [17.16-22.42]	9.83 [8.02-11.99]	19.65 [18.29-21.09]	9.7 [8.71-10.79]	20.27 [18.94-21.66]	12.36 [11.29-13.51]
Manipur	10.43 [7.09-15.1]	4.78 [2.67-8.43]	14.81 [11.69-18.57]	4.37 [2.77-6.83]	12.86 [10.51-15.66]	2.14 [1.27-3.59]	7.35 [6.32-8.52]	1.88 [1.39-2.55]	7.87 [6.44-9.58]	2.45 [1.7-3.52]
Meghalaya	18.31 [12.78-25.54]	12.68 [8.13-19.22]	16.1 [12.17-21.01]	9.36 [6.41-13.49]	15.64 [11.59-20.76]	25.1 [20.05-30.93]	16.91 [15.06-18.93]	6.44 [5.29-7.83]	15.25 [13.84-16.78]	8.3 [7.24-9.5]
Mizoram	9.77 [6.16-15.15]	4.6 [2.32-8.92]	11.81 [8.56-16.07]	4.51 [2.64-7.62]	9.65 [6.61-13.89]	3.09 [1.55-6.05]	7.33 [6.18-8.67]	1.77 [1.24-2.52]	7.96 [6.35-9.93]	3.81 [2.74-5.29]
Nagaland	12.5 [8.83-17.41]	3.45 [1.73-6.74]	11.69 [8.25-16.32]	7.66 [4.94-11.7]	13.74 [11.36-16.53]	4.97 [3.57-6.88]	8.65 [7.34-10.16]	3.19 [2.42-4.19]	14.44 [12.51-16.61]	7.88 [6.45-9.61]
Orissa	26.64 [23.62-29.89]	19.16 [16.52-22.11]	27.15 [24.26-30.25]	19.24 [16.73-22.04]	22.26 [19.18-25.68]	14.31 [11.78-17.27]	22.81 [21.5-24.17]	9.42 [8.53-10.4]	19.14 [17.8-20.54]	10.31 [9.3-11.42]
Punjab	23.15 [20-26.63]	12.86 [10.45-15.73]	14.26 [11.38-17.72]	6.17 [4.32-8.74]	15.28 [12.27-18.88]	7.64 [5.54-10.46]	14.36 [12.85-16.02]	6.57 [5.54-7.78]	12.83 [11.42-14.39]	5.65 [4.71-6.76]
Rajasthan	22.41 [19.69-25.39]	18.76 [16.23-21.57]	23.91 [21.82-26.12]	18.94 [17.04-21]	21 [18.21-24.09]	12.33 [10.15-14.91]	21.39 [20.38-22.45]	11.43 [10.65-12.25]	16.38 [15.42-17.39]	11.44 [10.62-12.32]
Sikkim			9.69 [6.63-13.95]	3.1 [1.56-6.08]	10.11 [6.54-15.3]	4.79 [2.51-8.94]	6.35 [4.32-9.22]	2.79 [1.55-4.97]	4.33 [2.27-8.11]	7.69 [4.77-12.19]
Tamil Nadu	25.92 [22.63-29.5]	14.72 [12.15-17.72]	19.51 [16.88-22.45]	9.63 [7.75-11.91]	18.58 [15.61-21.96]	4.17 [2.81-6.14]	16.16 [14.8-17.62]	6.72 [5.82-7.75]	13.58 [12.29-14.98]	7.37 [6.41-8.46]
Telangana	20.06 [16.11-24.7]	16.17 [12.6-20.51]	21.27 [17.1-26.14]	8.89 [6.21-12.57]	15.59 [13.05-18.51]	6.47 [4.85-8.58]	18.45 [16-21.18]	7.54 [5.96-9.5]	18.79 [17.36-20.3]	8.88 [7.86-10.01]
Tripura	19.71 [13.88-27.22]	18.25 [12.64-25.61]	19.59 [13.97-26.78]	11.49 [7.26-17.7]	18.81 [14.16-24.55]	13.76 [9.79-19]	14.54 [11.59-18.09]	7.05 [5.03-9.8]	14.53 [12.2-17.21]	9.16 [7.31-11.42]
Uttar Pradesh	26.72 [25.05-28.45]	26.45 [24.78-28.18]	23.79 [21.7-26.02]	22.12 [20.08-24.3]	22.11 [20.39-23.93]	14.82 [13.37-16.4]	23.49 [22.82-24.18]	12.07 [11.56-12.6]	17.22 [16.58-17.89]	12.22 [11.66-12.8]
Uttaranchal	23.28 [19.44-27.63]	17.89 [14.47-21.92]	21.93 [15.27-30.45]	14.04 [8.78-21.69]	18.42 [14.99-22.43]	9.81 [7.3-13.05]	15.15 [13.63-16.8]	7.93 [6.82-9.21]	11.14 [9.58-12.91]	7.93 [6.62-9.48]
West Bengal	29.74 [26.81-32.84]	22.7 [20.05-25.59]	26.77 [23.63-30.16]	13.31 [11-16.03]	20.47 [17.77-23.46]	11.14 [9.11-13.56]	20.47 [18.76-22.31]	8.17 [7.04-9.46]	18.97 [17.38-20.67]	10.2 [8.99-11.54]
**Union Territories (UTs)**										
Andaman & Nicobar Islands							12.26 [8.8-16.82]	3.83 [2.07-6.97]	13.66 [9.17-19.88]	10.56 [6.67-16.33]
Chandigarh							15.38 [8.48-26.29]	6.15 [2.33-15.28]	13.21 [6.43-25.21]	7.55 [2.86-18.45]
Dadra & Nagar Haveli and Daman & Diu							20.16 [15.59-25.68]	8.23 [5.37-12.41]	20.6 [16.17-25.87]	13.11 [9.56-17.71]
Jammu & Kashmir	16.93 [14.09-20.2]	12.09 [9.68-15.01]	17.76 [14.75-21.23]	7.48 [5.53-10.03]	13.02 [10.09-16.65]	8.85 [6.45-12.02]	8.95 [7.91-10.11]	4.28 [3.57-5.13]	12.81 [11.42-14.34]	10.92 [9.63-12.36]
Ladakh					13.02 [10.09-16.65]	8.85 [6.45-12.02]	8.98 [5.99-13.26]	4.08 [2.21-7.42]	17.22 [12.38-23.45]	13.33 [9.1-19.12]
Lakshadweep							18.42 [12.33-26.61]	5.26 [2.38-11.22]	18.56 [12.02-27.54]	13.4 [7.94-21.73]
NCT of Delhi	21.15 [18.4-24.19]	11.75 [9.65-14.23]	16.71 [13.32-20.75]	9.51 [6.97-12.85]	13.14 [9.63-17.68]	11.31 [8.07-15.64]	15.23 [12.21-18.84]	7.06 [5.04-9.82]	14.73 [12.63-17.1]	6.69 [5.28-8.45]
Puducherry							13.2 [10-17.22]	4.69 [2.89-7.52]	14.39 [10.73-19.02]	4.68 [2.73-7.89]

Table S3. Prevalence of Wasting (%) and 95% Confidence Intervals for the States/UTs of India, 1993-2021

	1993		1999		2006		2016		2021	
	Moderate	Severe	Moderate	Severe	Moderate	Severe	Moderate	Severe	Moderate	Severe
All India	15.01 [14.43-15.62]	8.42 [7.97-8.89]	13.18 [12.68-13.7]	7.59 [7.2-8]	14.49 [13.96-15.04]	8.49 [8.07-8.92]	14.71 [14.48-14.94]	9.63 [9.44-9.82]	12.26 [12.03-12.49]	9.38 [9.18-9.59]
**States**										
Andhra Pradesh			8.61 [6.12-11.99]	3.89 [2.32-6.46]	10.44 [8.36-12.97]	4.41 [3.1-6.24]	15.11 [13.09-17.38]	8.4 [6.88-10.21]	11.36 [9.53-13.48]	7.94 [6.41-9.79]
Arunachal Pradesh	12.18 [7.9-18.31]	8.33 [4.9-13.82]	5.09 [2.84-8.96]	4.63 [2.51-8.39]	10.39 [7.32-14.56]	6.81 [4.39-10.43]	8.58 [7.19-10.2]	7.38 [6.1-8.91]	6.3 [5.25-7.54]	6.93 [5.83-8.22]
Assam	11.44 [9.19-14.15]	3.13 [2.03-4.81]	9.44 [6.98-12.67]	10.9 [8.23-14.28]	11.7 [9.09-14.94]	7.45 [5.39-10.2]	11.06 [10.05-12.17]	6.5 [5.71-7.38]	12.33 [11.29-13.46]	10.68 [9.7-11.75]
Bihar	20.77 [18.54-23.19]	13.93 [12.06-16.04]	14.12 [12.2-16.29]	12.42 [10.61-14.49]	24.68 [21.89-27.69]	11.69 [9.69-14.03]	16.39 [15.64-17.17]	10.22 [9.61-10.86]	16.51 [15.67-17.38]	12.02 [11.29-12.78]
Chhattisgarh			19.54 [14.31-26.1]	6.9 [3.96-11.75]	17.4 [14.5-20.74]	9.84 [7.65-12.57]	17.05 [15.84-18.34]	10.04 [9.09-11.08]	13.18 [11.99-14.47]	10.68 [9.61-11.86]
Goa	12.32 [9.66-15.57]	5.22 [3.55-7.61]	11.96 [8.21-17.1]	5.74 [3.29-9.84]	9.93 [7.03-13.85]	6.29 [4.05-9.65]	10.64 [6.52-16.9]	10.64 [6.52-16.9]	15.5 [10.23-22.81]	10.08 [5.94-16.58]
Gujarat	16.05 [13.6-18.84]	11.01 [8.96-13.45]	14.2 [11.78-17.03]	8.35 [6.49-10.67]	11.59 [9.14-14.59]	7.85 [5.85-10.45]	18.74 [17.26-20.31]	13.86 [12.56-15.27]	14.86 [13.7-16.1]	12.97 [11.88-14.15]
Haryana	6.03 [4.55-7.96]	2.88 [1.91-4.34]	6.08 [4.39-8.35]	3.99 [2.67-5.94]	18.52 [15.22-22.34]	7.19 [5.16-9.94]	12.59 [11.41-13.88]	11.47 [10.34-12.71]	9.03 [7.94-10.25]	6.34 [5.43-7.39]
Himachal Pradesh			11.28 [8.87-14.23]	4.99 [3.44-7.18]	11.9 [8.85-15.83]	8.04 [5.57-11.46]	10.94 [9.18-12.99]	6.29 [4.96-7.95]	8.64 [7.04-10.56]	6.91 [5.48-8.67]
Jharkhand	11.94 [7.45-18.6]	2.99 [1.12-7.68]	15.93 [12.03-20.79]	16.3 [12.35-21.19]	21.67 [18.36-25.4]	16.35 [13.43-19.76]	19.88 [18.72-21.1]	14.53 [13.51-15.61]	16.31 [15.13-17.56]	11.48 [10.48-12.58]
Karnataka	20.34 [17.8-23.15]	8.51 [6.83-10.55]	18.65 [15.97-21.65]	10.08 [8.09-12.5]	14.74 [12.05-17.92]	6.22 [4.5-8.54]	15.55 [14.2-17]	10.65 [9.52-11.91]	12.94 [11.74-14.25]	9.61 [8.56-10.77]
Kerala	10.68 [8.6-13.19]	2.85 [1.85-4.37]	12.34 [9.42-16]	3.08 [1.76-5.35]	11.62 [8.73-15.31]	5.68 [3.73-8.55]	12.14 [10.09-14.54]	7.09 [5.53-9.05]	11.49 [9.58-13.72]	6.89 [5.42-8.73]
Madhya Pradesh			16.8 [14.84-18.96]	11.73 [10.07-13.62]	22.94 [20.5-25.59]	16.63 [14.5-19.02]	19.37 [18.54-20.22]	12.83 [12.14-13.56]	14.04 [13.16-14.97]	8.59 [7.89-9.35]
Maharashtra	19.1 [16.45-22.06]	13.53 [11.27-16.16]	16.3 [14.18-18.67]	7.57 [6.12-9.34]	12 [10.01-14.33]	7.77 [6.17-9.74]	17.98 [16.67-19.37]	10.4 [9.38-11.53]	14.93 [13.72-16.23]	12.16 [11.06-13.36]
Manipur	6.09 [3.64-10.01]	4.35 [2.36-7.89]	9.22 [6.78-12.42]	2.91 [1.66-5.06]	10.11 [8.02-12.66]	3.22 [2.11-4.88]	4.59 [3.79-5.55]	2.25 [1.7-2.96]	5.86 [4.63-7.39]	5.15 [4-6.61]
Meghalaya	11.97 [7.57-18.42]	8.45 [4.86-14.29]	10.49 [7.34-14.77]	6.37 [3.99-10]	11.93 [8.42-16.65]	23.05 [18.18-28.76]	9.98 [8.54-11.64]	9.7 [8.28-11.34]	9.11 [7.98-10.38]	6.39 [5.44-7.49]
Mizoram	5.17 [2.71-9.64]	1.15 [0.29-4.48]	8.33 [5.65-12.13]	4.86 [2.9-8.04]	6.18 [3.82-9.84]	4.63 [2.65-7.98]	5.32 [4.35-6.5]	2.96 [2.25-3.88]	5.09 [3.8-6.79]	5.56 [4.2-7.33]
Nagaland	6.9 [4.27-10.96]	3.45 [1.73-6.74]	8.47 [5.59-12.64]	7.26 [4.62-11.22]	11.55 [9.36-14.17]	5.56 [4.07-7.54]	7.28 [6.09-8.69]	3.9 [3.04-4.99]	11.12 [9.39-13.13]	6.62 [5.29-8.26]
Orissa	19.16 [16.52-22.11]	10.37 [8.39-12.74]	20.9 [18.29-23.77]	11.45 [9.48-13.78]	16.38 [13.68-19.48]	7.31 [5.52-9.63]	16.66 [15.5-17.88]	9.27 [8.38-10.23]	13.22 [12.06-14.48]	8.8 [7.84-9.87]
Punjab	15.43 [12.8-18.49]	5.95 [4.34-8.1]	6.6 [4.68-9.23]	2.98 [1.77-4.97]	8.52 [6.28-11.44]	3.71 [2.32-5.89]	12.24 [10.84-13.8]	6.25 [5.25-7.44]	9.01 [7.79-10.39]	5.65 [4.69-6.79]
Rajasthan	13.03 [10.9-15.51]	12.79 [10.67-15.25]	12.52 [10.94-14.28]	6.16 [5.05-7.49]	16.8 [14.27-19.67]	7.45 [5.77-9.58]	15.94 [15.03-16.88]	10.86 [10.1-11.67]	11.03 [10.19-11.92]	9.19 [8.42-10.01]
Sikkim			4.26 [2.38-7.53]	3.1 [1.56-6.08]	9.57 [6.12-14.68]	5.32 [2.89-9.6]	9.14 [6.66-12.41]	5.58 [3.7-8.33]	2.72 [1.14-6.36]	10.33 [6.68-15.62]
Tamil Nadu			17.2 [14.71-20.02]	8.22 [6.48-10.36]	12.85 [10.35-15.84]	12.15 [9.73-15.08]	12.45 [11.24-13.77]	10.16 [9.06-11.38]	9.28 [8.17-10.52]	7.16 [6.19-8.27]
Telangana			6.35 [4.13-9.64]	3.49 [1.94-6.19]	10.44 [8.36-12.97]	4.41 [3.1-6.24]	18.1 [15.67-20.81]	9.98 [8.15-12.16]	13.42 [12.14-14.82]	9.75 [8.65-10.99]
Tripura	16.79 [11.42-24]	5.84 [2.95-11.24]	13.51 [8.89-20.02]	6.76 [3.67-12.1]	12.39 [8.63-17.46]	8.26 [5.26-12.72]	11.45 [8.83-14.72]	11.23 [8.64-14.48]	8.92 [7.03-11.26]	8.07 [6.28-10.33]
Uttar Pradesh	17.89 [16.46-19.41]	11.06 [9.91-12.33]	13.94 [12.27-15.79]	7.71 [6.46-9.17]	14.49 [13.05-16.06]	8.71 [7.58-9.99]	15.28 [14.71-15.86]	9.12 [8.67-9.59]	11.86 [11.29-12.45]	9.9 [9.37-10.44]
Uttaranchal	12.99 [10.06-16.61]	6.37 [4.37-9.19]	9.65 [5.42-16.59]	1.75 [0.44-6.74]	13.88 [10.88-17.53]	4.07 [2.54-6.44]	11.64 [10.3-13.14]	11.13 [9.82-12.6]	9.18 [7.74-10.86]	7.13 [5.86-8.65]
West Bengal			13.88 [11.52-16.63]	5.52 [4.06-7.47]	13.47 [11.24-16.07]	6.87 [5.28-8.88]	14.12 [12.66-15.72]	8.57 [7.42-9.89]	12.23 [10.88-13.72]	6.85 [5.83-8.03]
**Union Territories (UTs)**										
Andaman & Nicobar Islands							8.05 [5.3-12.02]	5.75 [3.49-9.31]	14.38 [9.57-21.06]	4.79 [2.3-9.71]
Chandigarh							12.31 [6.28-22.73]	10.77 [5.22-20.91]	7.55 [2.86-18.45]	5.66 [1.84-16.13]
Dadra & Nagar Haveli and Daman & Diu							13.17 [9.47-18.03]	8.64 [5.7-12.89]	18.26 [13.87-23.65]	4.15 [2.25-7.54]
Jammu & Kashmir	11.74 [9.36-14.63]	6.04 [4.37-8.3]	12.15 [9.64-15.2]	5.61 [3.95-7.91]	14.25 [11.18-17.99]	6.63 [4.59-9.5]	8.21 [7.22-9.34]	5.71 [4.88-6.67]	11.13 [9.75-12.68]	11.64 [10.23-13.22]
Ladakh					14.25 [11.18-17.99]	6.63 [4.59-9.5]	4.49 [2.5-7.92]	3.67 [1.92-6.91]	6.45 [3.51-11.57]	12.9 [8.48-19.16]
Lakshadweep							11.4 [6.74-18.65]	3.51 [1.32-8.98]	8.79 [4.46-16.61]	13.19 [7.64-21.8]
NCT of Delhi	12.92 [10.73-15.49]	4.44 [3.19-6.15]	9 [6.53-12.27]	8.48 [6.09-11.69]	8.39 [5.64-12.31]	11.31 [8.07-15.64]	14.13 [11.21-17.65]	8.83 [6.54-11.82]	8.03 [6.44-9.96]	7.27 [5.76-9.13]
Puducherry							9.68 [6.96-13.3]	10.26 [7.46-13.96]	8.78 [5.9-12.86]	6.87 [4.37-10.64]

Table S4. Analytical Samples for Stunting, Underweight, and Wasting

Survey Year	Total Children (0-23 months)	Stunting		Underweight		Wasting	
		Missing or Implausible	Final Analytical Sample	Missing or Implausible	Final Analytical Sample	Missing or Implausible	Final Analytical Sample
1993	23,467	9,580	13,887	5,014	18,453	9,580	13,887
1999	19,269	2,512	16,757	2,512	16,757	2,512	16,757
2006	21,877	5,391	16,486	5,391	16,486	5,391	16,486
2016	105,863	15,549	90,314	15,549	90,314	15,549	90,314
2021	88,932	7,988	80,944	4,475	84,457	9,763	79,169

Table S5. Analytical Samples by State/Union Territory for Stunting

State / Union Territory	1993	1999	2006	2016	2021
Andaman & Nicobar Islands	0	0	0	261	151
Andhra Pradesh	0	360	680	1072	1008
Arunachal Pradesh	156	216	279	1341	1786
Assam	638	413	470	3371	3626
Bihar	1170	1119	847	9035	7440
Chandigarh	0	0	0	65	52
Chhattisgarh	0	174	569	3495	2946
Dadra & Nagar Haveli and Daman & Diu	0	0	0	243	244
Goa	479	209	302	141	133
Gujarat	754	683	535	2503	3508
Haryana	763	576	459	2772	2392
Himachal Pradesh	0	541	336	1033	1004
Jammu & Kashmir	579	535	407	2593	1826
Jharkhand	134	270	526	4316	3621
Karnataka	870	724	563	2572	2835
Kerala	702	389	370	832	925
Ladakh	0	0	0	245	245
Lakshadweep	0	0	0	114	95
Madhya Pradesh	0	1262	1046	8587	5760
Maharashtra	754	1043	875	3114	3180
Manipur	230	412	653	2178	1119
Meghalaya	142	267	243	1443	2226
Mizoram	174	288	259	1692	841
NCT of Delhi	766	389	274	453	935
Nagaland	232	248	684	1538	1102
Orissa	762	847	629	3788	3094
Puducherry	0	0	0	341	269
Punjab	622	470	458	1887	1884
Rajasthan	821	1510	738	6030	5178
Sikkim	0	258	188	394	201
Tamil Nadu	0	779	576	2618	2398
Telangana	0	315	680	862	2564
Tripura	137	148	218	454	733
Uttar Pradesh	2594	1492	2112	14981	12296
Uttaranchal	408	114	418	1967	1338
West Bengal	0	706	772	1983	2077

Table S6. Analytical Samples by State/Union Territory for Underweight

State / Union Territory	1993	1999	2006	2016	2021
Andaman & Nicobar Islands	0	0	0	261	161
Andhra Pradesh	495	360	680	1072	1031
Arunachal Pradesh	156	216	279	1341	1884
Assam	638	413	470	3371	3856
Bihar	1170	1119	847	9035	7724
Chandigarh	0	0	0	65	53
Chhattisgarh	262	174	569	3495	3094
Dadra & Nagar Haveli and Daman & Diu	0	0	0	243	267
Goa	479	209	302	141	136
Gujarat	754	683	535	2503	3663
Haryana	763	576	459	2772	2476
Himachal Pradesh	678	541	336	1033	1040
Jammu & Kashmir	579	535	407	2593	2014
Jharkhand	134	270	526	4316	3742
Karnataka	870	724	563	2572	2977
Kerala	702	389	370	832	962
Ladakh	0	0	0	245	180
Lakshadweep	0	0	0	114	97
Madhya Pradesh	1291	1262	1046	8587	5967
Maharashtra	754	1043	875	3114	3375
Manipur	230	412	653	2178	1144
Meghalaya	142	267	243	1443	2301
Mizoram	174	288	259	1692	892
NCT of Delhi	766	389	274	453	971
Nagaland	232	248	684	1538	1129
Orissa	762	847	629	3788	3172
Puducherry	0	0	0	341	278
Punjab	622	470	458	1887	1948
Rajasthan	821	1510	738	6030	5409
Sikkim	0	258	188	394	208
Tamil Nadu	625	779	576	2618	2497
Telangana	334	315	0	862	2704
Tripura	137	148	218	454	764
Uttar Pradesh	2594	1492	2112	14981	12790
Uttaranchal	408	114	418	1967	1374
West Bengal	881	706	772	1983	2177

Table S7. Analytical Samples by State/Union Territory for Wasting

State / Union Territory	1993	1999	2006	2016	2021
Andaman & Nicobar Islands	0	0	0	261	146
Andhra Pradesh	0	360	680	1072	995
Arunachal Pradesh	156	216	279	1341	1747
Assam	638	413	470	3371	3511
Bihar	1170	1119	847	9035	7306
Chandigarh	0	0	0	65	53
Chhattisgarh	0	174	569	3495	2883
Dadra & Nagar Haveli and Daman & Diu	0	0	0	243	241
Goa	479	209	302	141	129
Gujarat	754	683	535	2503	3384
Haryana	763	576	459	2772	2381
Himachal Pradesh	0	541	336	1033	984
Jammu & Kashmir	579	535	407	2593	1770
Jharkhand	134	270	526	4316	3544
Karnataka	870	724	563	2572	2758
Kerala	702	389	370	832	914
Ladakh	0	0	0	245	155
Lakshadweep	0	0	0	114	91
Madhya Pradesh	0	1262	1046	8587	5655
Maharashtra	754	1043	875	3114	3108
Manipur	230	412	653	2178	1126
Meghalaya	142	267	243	1443	2207
Mizoram	174	288	259	1692	845
NCT of Delhi	766	389	274	453	922
Nagaland	232	248	684	1538	1088
Orissa	762	847	629	3788	3010
Puducherry	0	0	0	341	262
Punjab	622	470	458	1887	1876
Rajasthan	821	1510	738	6030	5051
Sikkim	0	258	188	394	184
Tamil Nadu	0	779	576	2618	2360
Telangana	0	315	680	862	2481
Tripura	137	148	218	454	706
Uttar Pradesh	2594	1492	2112	14981	11934
Uttaranchal	408	114	418	1967	1318
West Bengal	0	706	772	1983	2044
